# Application of Improved Satin Bowerbird Optimizer in Image Segmentation

**DOI:** 10.3389/fpls.2022.915811

**Published:** 2022-05-06

**Authors:** Linguo Li, Shunqiang Qian, Zhangfei Li, Shujing Li

**Affiliations:** ^1^School of Computer and Information Engineering, Fuyang Normal University, Fuyang, China; ^2^School of Computer, Nanjing University of Posts and Telecommunications, Nanjing, China

**Keywords:** satin bowerbird optimizer, chaotic initialization, Cauchy mutation strategy, medical image, plant image

## Abstract

Aiming at the problems of low optimization accuracy and slow convergence speed of Satin Bowerbird Optimizer (SBO), an improved Satin Bowerbird Optimizer (ISBO) based on chaotic initialization and Cauchy mutation strategy is proposed. In order to improve the value of the proposed algorithm in engineering and practical applications, we apply it to the segmentation of medical and plant images. To improve the optimization accuracy, convergence speed and pertinence of the initial population, the population is initialized by introducing the Logistic chaotic map. To avoid the algorithm falling into local optimum (prematurity), the search performance of the algorithm is improved through Cauchy mutation strategy. Based on extensive visual and quantitative data analysis, this paper conducts a comparative analysis of the ISBO with the SBO, the fuzzy Gray Wolf Optimizer (FGWO), and the Fuzzy Coyote Optimization Algorithm (FCOA). The results show that the ISBO achieves better segmentation effects in both medical and plant disease images.

## Introduction

Image segmentation is an important means of image processing, analysis, understanding and computer vision. In addition to efficiently locating the region of interest in an image, it can also be widely used in the fields of feature extraction (Dobreva et al., 2021), image classification (Houssein et al., 2021; Xue et al., 2022), medical diagnosis (Kurmi et al., 2021) and plant disease segmentation (Akay et al., 2021). The quality of image segmentation is affected by many factors such as illumination change, inter-class difference and background complexity. There is still a considerable gap between existing image segmentation technology and the requirements of intelligent recognition and machine vision. Therefore, image segmentation remains a very open research field with unlimited potential. In recent times, a variety of image segmentation schemes have been proposed based on the needs of image understanding and machine vision, including thresholding, edge detection, and region methods (Pare et al., 2020; Mittal et al., 2021). The thresholding method divides the image into non-overlapping regions according to the gray level of the image through a threshold vector (one or more thresholds). Compared with other kinds of methods, such a method is easier to perform and involves less calculation. Therefore, it has been widely used in many fields, such as those involving medical images (Avola et al., 2021) and plant disease images (Neupane and Baysal Gurel, 2021).

Although the thresholding method is a simple and effective image segmentation procedure, the selection of optimal threshold remains crucial due to the influence of image background complexity, regional contrast and other factors. With the increase in the number of thresholds, the amount of calculation involved in this method also increases considerably. Therefore, the intelligent optimization algorithm based on specific objective function is widely used (Singh et al., 2021). Yazid et al. (2022) comprehensively analyze the effects of image brightness difference, target size and noise on Kapur and Li entropies in image thresholding. Through numerous experiments, the author verify the effectiveness of the two entropies, and put forward reasonable parameter setting suggestions for the application of the two entropies in image segmentation. Wu et al. (2021) take Kapur as the objective function and improve the preconditioning optimization algorithm (HPOA) through the evolutionary state strategy. In the experimental verification stage, the authors verify the excellent performance of Kapur entropy in color image segmentation through peak signal-to-noise ratio (PSNR), feature similarity index (FSIM) and structural similarity index (SSIM); this method is superior to other methods such as the moth flame optimization algorithm (MFO), and the multiverse optimizer (MVO). Sowjanya and Injeti (2021) verify the effects of Kapur and Otsu objective functions in multi-thresholding image segmentation through the butterfly optimization algorithm (BOA) and the gasses Brownian motion optimization (GBMO) fusion algorithm. Compared with seven optimization algorithms such as WOA (whale optimization algorithm) and SSO (social spider optimization), this method obtains better optimal threshold and further improves the image segmentation quality. Liu et al. (2021) use 2D Kapur entropy as the objective function and improve the differential evolution (DE) algorithm through slime mould foraging behavior, verifying the better performance of this method in breast cancer image segmentation. Experimental results show that this method not only improves convergence accuracy, but also reduces the risk of falling into local optimization. Kurban et al. (2021) use six intelligent optimization algorithms including equilibrium optimization (EO) and political optimizer (PO) to optimize Kapur and Otsu objective functions and obtain the optimal threshold. Through the evaluation indices such as PSNR, FSIM, Kapur-based marine predictors algorithm (MPA) and turbine flow of water-based optimization (TFWO) perform better than the other four methods in color aerial image segmentation. Anitha et al. (2021) take Kapur and Otsu as the objective function and obtain the optimal threshold through the modified white optimization algorithm (MWOA). Compared with GA, PSO and ABC algorithms, this method demonstrates superior convergence speed, feature stability and image segmentation quality. The time efficiency of this method is also higher. According to the specific objective function-based multi-level image thresholding methods in recent years, the use frequency of Kapur entropy is relatively high, and it also shows a reliable optimization effect in experimental comparison. Therefore, this paper continues to take the fuzzy Kapur (Li et al., 2016) as the objective function to analyze the performance of intelligent optimization algorithms in medical and plant image segmentation.

In view of the high computational complexity of multi-level thresholding and the demanding requirements of medical and plant image segmentation, the intelligent optimization algorithm is widely used to obtain the optimal threshold (Avola et al., 2021; Mittal et al., 2021; Neupane and Baysal Gurel, 2021). Li et al. (2016) improve the search strategy of gray wolf optimizer (GWO) by weighting the optimal population, enhancing the quality of image segmentation through median aggregation. The experimental verification of Berkeley segmentation dataset benchmarks 500 (BSD500) shows that this method obtains higher image segmentation accuracy in evaluation parameters such as PSNR and FSIM, compared with GWO, electromagnetism optimization (EO) and fuzzy DE. Su et al. (2022) improve the artificial bee colony algorithm (ABC) through horizontal and vertical search strategies. This algorithm improves the convergence speed and the quality of the optimal solution of ABC to some extent. In the experimental verification, the authors compare the performance of the algorithm with that of the original algorithm through 30 benchmark functions and apply the improved algorithm to Covid-19 X-ray images. In comparison with WOA, Sine Cosine Algorithm (SCA), Harris Hawks optimizer (HHO), Spherical Search optimizer (SSO) and other methods, the algorithm has better image segmentation effect on Covid-19 X-ray images with Kapur entropy as the objective function. Chen et al. (2021) improve the slim mount algorithm (SMA) through the threshold update mechanism of ABC and form a new ASMA fusion algorithm. Based on the analysis of 30 standard functions, this method obtains a better optimal solution and can effectively avoid prematurity. And the authors have achieved good results in the segmentation of standard images and lupus nephritis images. Li et al. (2021) inspired by the idea of differential evolution and make full use of the current number of iterations and the maximum number of iterations to improve the population search strategy of Coyote optimization algorithm (COA). Compared with COA, fuzzy ABC and fuzzy GWO, this method is better in visual and PSNR segmentation quality evaluations of BSD500 and medical image segmentation. Chakraborty and Mali (2022) improve the cuckoo search (CS) algorithm using fuzzy theory. Through the evaluation indices such as PSNR, SSIM, this method enjoys certain advantages in medical image segmentation quality and calculation time, compared with the improved DE, moth flame optimization algorithm and other methods. Singh et al. (2022) combine the tune swarm algorithm (TSA) and the naked mole rat algorithm (NMRA), and compare the data of CEC 2019 standard function and image segmentation, they show that this method is better than PSO, GA and others. According to the application of intelligent optimization algorithms in multi-level thresholding in recent years, we find this kind of method with numerous variations has been used widely in different fields. Taking account of the characteristics of medical and plant images, and the amount of experimental verification involved, this paper seeks to enhance the initialization and optimal population search strategy of the SBO. It further seeks to refine its optimization accuracy and convergence speed in image segmentation. Thus, image segmentation quality is improved.

The remainder of this paper is set out as follows: “Overview of the SBO” section briefly introduces the SBO. “The Improved Satin Bowerbird Optimizer Based on Chaos Initialization and Cauchy Mutation” section presents the improved SBO (ISBO) based on chaotic initialization and Cauchy mutation strategy. “Selection of the Objective Function” section analyzes the selection of objective function corresponding to ISBO. “Comparison and Analysis of Experimental Results with the Medical and Plant Images” section details the performance of ISBO for medical and plant image segmentation. Finally, “Conclusion” section concludes this study.

## Overview of the SBO

Satin Bowerbird Optimizer (SBO; Moosavi and Bardsiri, 2017) is an intelligent optimization algorithm that simulates the breeding behavior of an adult male Satin Bowerbird in the wild. As a wild bird with strong survival and reproduction skills, a mature male Satin Bowerbird wins the favor of the female by carefully constructing a courtship cabin, and attracts the opposite sex through continuous loud singing, holding a luminous object in its beak, to improve the probability of a successful courtship. In the courtship process the male should not only ensure a successful courtship cabin construction, but also constantly resist the challenges of its competitors, to prevent the nest from being damaged. According to Satin Bowerbird survival “rules,” the SBO algorithm includes the following steps:

1. Random generation of the initial population of Satin Bowerbirds. An initial population of several *NB* individuals is randomly generated in a solution space. The position of each courtship cabin is defined as *D*-dimension, and the current population evolution algebra is *t*.
2. Calculate the fitness value (objective function) of each individual, and then calculate the ratio of the fitness value to the overall fitness value to represent the probability of the individual being selected. The probability of selecting the courtship cabin is calculated by Equation (1), ${\mathrm{fit}}_{i}$ represents the fitness value of the i-th courtship cabin, which can be calculated by Equation (2), $f\left(x_{i}\right)$ represents the objective function value of the i-th courtship cabin.
  (1) $$\mathrm{Pro}b_{i}=\frac{fit_{i}}{\sum_{n=1}^{NB}fit_{n}}$$
  (2) $$fit_{i}=\{\begin{array}{l} \frac{1}{1+f\left(x_{i}\right)},f\left(x_{i}\right)\geq 0\\ 1+|f\left(x_{i}\right)|,f\left(x_{i}\right)<0 \end{array}$$
3. Update the population. According to the position information of the last iteration, the male Satin Bowerbird constantly adjusts the position of the courtship cabin, to continuously approach the optimal solution. The position update formula is as follows.
(3) $$x_{ik}^{t+1}=x_{ik}^{t}+\lambda_{k}\left(\left(\frac{x_{jk}+x_{\mathrm{elite},k}}{2}\right)-x_{ik}^{k}\right)$$
  Where $x_{ik}^{t}$ is the k-dimensional component of the i-th individual in the t-th iteration; $x_{jk}$ is the k-dimensional component of the optimal position ever found at present, $x_{j}$is determined by the roulette selection mechanism; $x_{\mathrm{elite},k}$ is the k-dimensional component of the current global optimal position of the whole population. $\lambda_{k}$ is the step factor, which is calculated by equation (4).
(4) $$\lambda_{k}=\frac{\alpha }{1+P_{j}}$$
  Where α is the maximum step size, and $P_{j}$ is the probability of selecting the target courtship cabin, $P_{j}\in \left[0,1\right]$. Equation (4) clearly shows that the greater the probability of selecting the target location, the smaller the step size. When the probability of selecting the target location is 0, the step size is the largest, denoted as α. When the probability of selecting the target location is 1, the step size is the smallest, denoted as α/2.
4. Individual variation to prevent it from falling into local optimization. A strong male often steals from other males’ courtship cabins, and even destroys those cabins. Therefore, at the end of each iteration of the algorithm, there is a certain random mutation probability to improve the mutation of the algorithm. At this stage, $x_{ik}$ the follows the normal distribution, as shown in Equation (5).
(5) $$x_{ik}^{t+1}∼N\left(x_{ik}^{t},\sigma^{2}\right)$$
(6) $$N\left(x_{ik},\sigma^{2}\right)=x_{ik}^{t}+\left(\sigma *N\left(0,1\right)\right)$$
  The calculation formula of the SD is as:
(7) $$\sigma =z*\left(\;{\mathrm{var}}_{\text{max}}-{\mathrm{var}}_{\text{min}}\right)$$
  Where *z* is the scaling factor, ${var}_{\text{max}}$ and ${var}_{\text{min}}$ are, respectively, the upper and lower limits of the variable$x_{i}$.
5. At the end of each iteration, a new combination population is formed from the initial population and the population obtained from the mutation, and the fitness values of all individuals in the combination population are arranged in an ascending order. The individual with the largest objective function value is retained, and the other individuals are removed. If the end condition is satisfied at this stage, the optimal position and its corresponding optimal value will be outputted. Otherwise, the iteration will continue until the maximum number of iterations is reached.

## The Improved Satin Bowerbird Optimizer Based on Chaos Initialization and Cauchy Mutation

### Initialization of Logistic Chaos

Although according to the natural law, the initial population of the intelligent optimization algorithm adopts a random initialization mode, based on the purpose of the engineering application and convergence speed requirements, a better initialization method will greatly accelerate the convergence speed of intelligent optimization algorithm. The SBO also uses random values to initialize the population. Therefore, in this paper we introduce a Logistic chaotic map (Aniszewska, 2018) to improve the diversity of the initial population, thus obtaining a better initial population, and finally improving the optimization accuracy and convergence speed of the algorithm. The calculation method of Logistic chaotic map is shown in Equation (8):


(8) $$X_{i+1}=\mu X_{i}.*\left(1-X_{i}\right)$$

The value range of control parameters μ is set from 0 to 4. When the value of μ is larger, its chaos will be stronger. When the value of μ is 4, the chaotic initialization effect will be enhanced. Therefore, we take the value of μ at 4. Thus, the population initialization equation can be changed to Equation (9):


(9) $$\mathrm{pop}\;\left(i\right).\mathrm{Position}=Z\;\left(i,:\right).*\left(\mathrm{VarMax}-\mathrm{VarMin}\right)+\mathrm{VarMin}$$

Where $Z\left(i,:\right)$ represents $X_{i+1}$ of Equation (8).

### Cauchy Variation Strategy

The SBO is prone to fall into local optimization in its mutation stage. To solve this problem, this paper uses the Cauchy mutation strategy (Karakus et al., 2020) to replace the original mutation strategy of the SBO. The peak distribution of Cauchy function at the coordinate origin is shorter, but the distribution in the rest is longer. Using Cauchy mutation can produce greater disturbance nearby the current population. Compared with the original mutation strategy of the SBO, the improved method can produce greater and wider individual mutation, thus ensuring the flexibility and distinctiveness of mutation. The calculation equation of Cauchy variation strategy is shown in Equation (10):


(10) $$X_{i,j}^{t+1}=X_{\mathrm{best}}\left(t\right)+\mathrm{Cauchy}\left(0,1\right)⊕X_{\mathrm{best}}\left(t\right)$$

Where *Cauchy* (0,1) represents the standard Cauchy distribution; $X_{\mathrm{best}}\left(t\right)$ is the position of an individual that needs variation.

The corresponding variation probability is calculated by Equation (11):


(11) $$P_{s}=-\exp{\left(1-\frac{\mathrm{it}}{\mathrm{MaxIt}}\right)}^{20}+p$$

Where the value of *p* is taken at 0.05, MaxIt represents the maximum number of iterations, and it represents the current number of iterations. If *r* and <*Ps*, the Cauchy mutation operation will not be performed. Otherwise, the mutation operation will continue.

## Selection of the Objective Function

According to the analysis of “Introduction”section, Kapur entropy is the commonly used objective function of multi-level thresholding. In Li et al. (2021), the Kapur entropy based on fuzzy theory is proposed, and the experiments show that the fuzzy Kapur entropy has a better optimization effect. Therefore, in order to reflect the optimal performance of the ISBO, the fuzzy Kapur continues to be set as the objective function in our work; next the optimal threshold is obtained through ISBO optimization, and the image is divided into multiple target regions. Assuming $I\left(x,y\right)$t is the gray image that needs to be processed, ${th}_{l},{th}_{2},\ldots ,{th}_{d}$ are the preset D thresholds, then fuzzy Kapur represents the sum of probability statistics being divided into D + 1 different gray distributions, which are shown in Equation (12):


(12) $$H\left(th_{1},th_{2},\ldots ,th_{d}\right)=H_{0}+H_{1}+\ldots +H_{d}$$

Where $H_{i}$ is the entropy of the i-th gray distribution, which is expressed as:


(13) H0=−∑i=0L−1μ0i∗piω0lnμ0i∗piω0ω0=∑i=0L−1μ0i∗piH1=−∑i=0L−1μ1i∗piω1lnμ1i∗piω1ω1=∑i=0L−1μ1i∗piHj=−∑i=0L−1μji∗piωjlnμji∗piωjωj=∑i=0L−1μji∗piHm=−∑i=0L−1μmi∗piωmlnμmi∗piωmωm=∑i=0L−1μmi∗pi

Where $\mu_{0}\left(i\right)$ is trapezoidal membership function (Li et al., 2016), and the threshold is calculated by fuzzy parameters, as shown in Equation (14):


(14) $$th_{1}=\frac{a_{1}+a_{2}}{2},th_{2}=\frac{a_{3}+a_{4}}{2},\cdots ,th_{d}=\frac{a_{n-1}+a_{n}}{2}$$

## Comparison and Analysis of Experimental Results With the Medical and Plant Images

### Parameter Setting and Discussion

In order to verify the value of ISBO in engineering and practical applications, this paper focuses on the segmentation effect in medical and plant images. Medical image segmentation can assist doctors to determine the location of lesions, especially in patients with mild disease, and can effectively help avoid doctors’ diagnostic errors. Plant disease segmentation also has high application value in the field of agriculture. It can effectively detect pests and diseases and improve crop yield in a timely manner, especially in large-scale planting. This section first analyzes and compares the segmentation effects of medical images, and then gives the experimental results of plant images in the following section. The experiment of ISBO is performed on Windows10 (64bit), Intel Core i5 processor with 8GB RAM, using programming software MATLAB R2016a. The setting of the experimental parameters is shown in Table 1, like in Moosavi and Bardsiri (2017) and Li et al. (2021).

**Table 1:** Experimental parameter setting.

Parameter	nPop	Alpha	Thresholds	Iterations
Value	20	0.94	2,3,5	1,000

**Figure 1:**
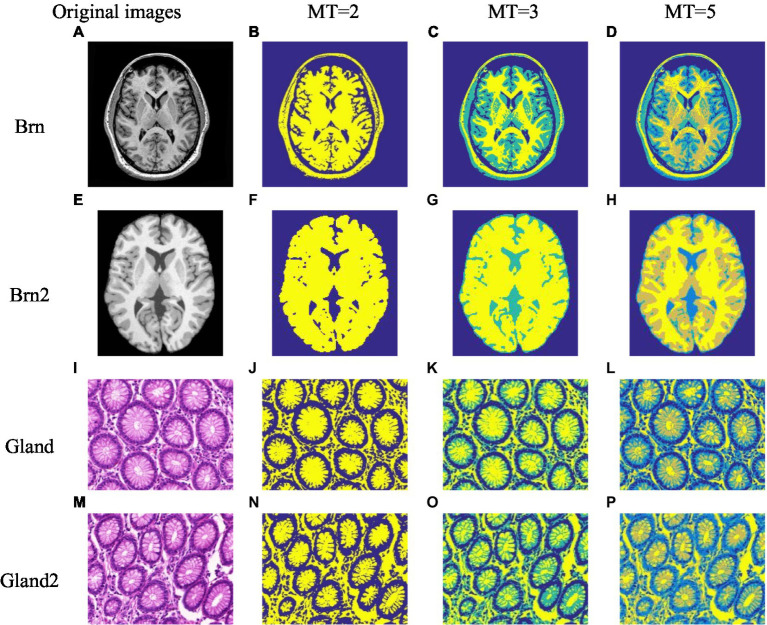
The medical image segmentation results based on the ISBO. **(A-P)** only characterize the serial numbers of different experimental images.

In order to prove that the parameter setting in Table 1 can improve the optimization effect of ISBO, Table 2 lists the PSNR and FSIM image segmentation quality evaluation values of Brn (Figure 1A) images with different population numbers, when the threshold is 5. According to the literature review in “Introduction” section, PSNR and FSIM are the two most commonly used evaluation indices in image segmentation. PSNR is called peak signal-to-noise ratio. The greater its value, the smaller the distortion of the image. FSIM is called feature similarity. The greater its value, the higher the similarity between the two images, and the better the quality of image segmentation. It can be observed from Table 2 that when the population number is 20, the PSNR value and the FSIM value reach their maximum. With the increase in the population number, the values of PSNR and FSIM begin to decrease gradually. Therefore, in this paper, the population number of 20 is adopted.

**Table 2:** Experimental results with different population numbers.

nPop	20	40	50	60	80
PSNR	26.1277	25.8615	25.4419	25.3696	24.9538
FSIM	0.9069	0.8951	0.8910	0.8890	0.8575

In (Moosavi and Bardsiri, 2017), the step size is set to 0.94. In order to verify whether this step size is optimal, Table 3 lists the impact of step threshold on image segmentation quality with the same image. Table 3 demonstrates that when the value of the step threshold increases from 0.5 to 0.94, the PSNR value and FSIM value also increase. However, when the step threshold continues to increase from 0.94 onwards, the PSNR value and FSIM value begin to decrease. Then we can determine that the result is optimized, when the maximum step is 0.94. Therefore, this paper sets the threshold step to 0.94.

**Table 3:** Experimental results with different values of the step thresholds.

Alpha	0.5	0.94	1	1.5
PSNR	23.8823	26.1277	25.9621	24.8653
FSIM	0.8621	0.9069	0.8966	0.8593

Table 4 lists the effects of the maximum number of iterations on image segmentation quality. When the maximum number of iterations is 1,000, the PSNR value and FSIM value of the algorithm in this paper are optimal, therefore we decide to set the maximum number of iterations of ISBO to 1,000.

**Table 4:** Experimental results with different maximum iterations.

Iterations	500	1,000	3,000	5,000	10,000
PSNR	25.1937	26.1277	24.4850	25.1867	25.5288
FSIM	0.8604	0.9069	0.8547	0.8912	0.8791

**Figure 2:**
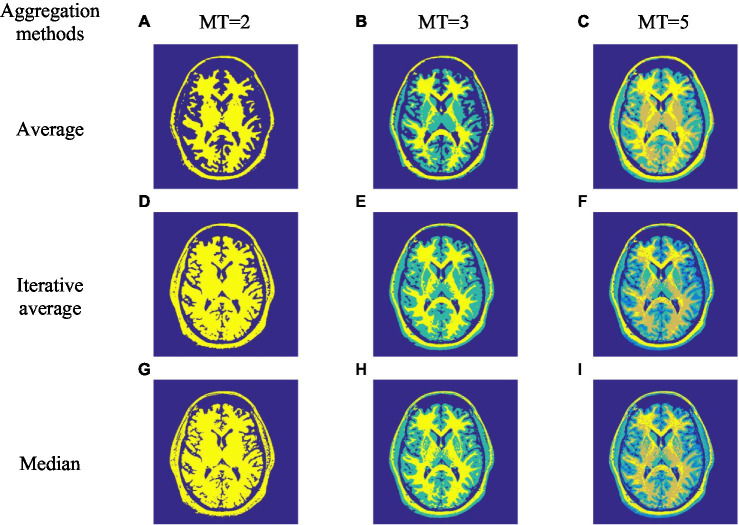
Image segmentation effect of improved satin bowerbird optimizer (ISBO) based on three aggregation methods. **(A-I)** only characterize the serial numbers of different experimental images.

Three spatial local information aggregation methods are cited in Li et al. (2016, 2021). Similarly, this paper also uses regional aggregation to improve the effect of image segmentation. To further compare the advantages and disadvantages of the proposed algorithm where the three aggregation methods are applied, Figure 2 shows the segmentation results of Brn (Figure 1A) image by ISBO algorithm using the three aggregation methods, and Table 5 lists the PSNR evaluation value obtained by using the proposed method in this paper under different thresholds.

**Table 5:** Experimental results with three different aggregation methods.

No. of thresholds	PSNR		
	Average	Iterative median	Median
2	15.6635	18.1473	18.2564
3	18.7338	22.5321	22.6985
5	25.8563	26.0101	26.2851

Figures 2A–I demonstrates that the three aggregation methods have achieved satisfactory image segmentation results, but the visual contrast effect is not visibly obvious. In order to demonstrate the advantages and disadvantages of the three methods in detail, we can observe from Table 5 that the median aggregation method obtains higher PSNR values under all thresholding conditions, compared with the other two methods. Therefore, ISBO is combined with the median aggregation method to complete the next experimental comparison and analysis.

### Medical Image Experiment Results

Figures 1B–D, F–H, J–L and N–P show the image segmentation results of four medical images [Brain images: (A) and (E), Gland images: (I) and (M)] which collected from Simulated Brain Database (SBD)^1^ and Gland Segmentation in Colon Histology Images (GlaS)^2^ with different threshold numbers. In order to more intuitively demonstrate the quality of image segmentation results, Table 6 lists the threshold distribution, PSNR and FSIM of the four medical images with different threshold numbers.

**Table 6:** The experimental result data with medical images.

Image	MT	Thresholds					PSNR	FSIM
Brn	2	38.5	169.5				18.2564	0.6299
	3	25.5	117	196			22.6985	0.7868
	5	15.5	76	137	162	221.5	26.2851	0.9080
Brn2	2	71.5	175.5				17.6193	0.5881
	3	26.5	86	211.5			20.6821	0.6828
	5	21	69	104	160	219.5	26.2558	0.8210
Gland	2	111.5	175				13.3887	0.5657
	3	77.5	150.5	183			13.8454	0.7133
	5	59.5	135.5	156.5	195	236.5	14.1263	0.8328
Gland2	2	93.5	189.5				13.2126	0.5746
	3	70	142.5	205.5			13.8357	0.7349
	5	24	105.5	136.5	172	243.5	14.1201	0.8404

As can be observed from Figure 1, for brain medical images and gland medical images, the ISBO algorithm can clearly segment the structures or tissues of different medical images, can effectively assist doctors in medical diagnosis, and can provide more refined pre-processing data for accurate diagnosis or disease prediction. In order to further compare it with other similar algorithms, as visual results alone cannot fully illustrate the problem, Table 6 lists the threshold distribution and PSNR and FSIM evaluation data of the images in Figure 1 after being optimized by the ISBO. From the data analysis, we can see that with the increase in the number of thresholds, the threshold distribution of the proposed method in our work tends to be balanced gradually, and better expected values are obtained in the PSNR and FSIM indices.

### Quantitative Comparison and Analysis of Similar Algorithms

To fully illustrate the advantages of the proposed algorithm in this paper, based on Figure 1; Tables 6, 7 lists the experimental data of ISBO and SBO, FGWO and FCOA, and analyzes the advantages and disadvantages of ISBO with PSNR as the evaluation standard.

**Table 7:** The experimental data with different algorithms.

Image	MT	PSNR			
		ISBO	SBO	FGWO	FCOA
Brn	2	18.2564	18.1473	18.1473	16.5122
	3	22.6985	22.5213	22.4268	21.4169
	5	26.2851	25.5405	25.5976	25.5721
Brn2	2	17.6193	17.5533	11.8691	13.5127
	3	20.6821	20.2781	19.5746	20.5012
	5	26.2558	25.1938	23.8653	25.2699
Gland	2	13.3887	13.2855	13.3207	13.3219
	3	13.8454	13.5825	13.7803	13.6153
	5	14.1263	14.1256	14.0953	14.0777
Gland2	2	13.2126	13.1912	13.2104	13.2140
	3	13.8357	13.6966	13.7586	13.7032
	5	14.1201	14.1110	14.1163	14.1113

The experimental data in Table 7 demonstrates that compared with SBO, ISBO obtains higher PSNR value in all cases. Specifically, the PSNR value of ISBO algorithm increases by 0.2583 on average, − a mean percentage increase of 1.47%. The maximum increase stands at 1.062 and the minimum increase at 0.0007. Similarly, the maximum percentage increase is 6.03%, the minimum 0.004%. Compared to the FGWO, the PSNR value of the ISBO is also superior with an average increase of 0.8803 and an average increase ratio of 5.18%, of which the maximum increase is 5.7502 and the minimum 0.0022. By percentage, the maximum increase is 33.86% and the minimum 0.01%. Finally, compared with the FCOA, the ISBO is better than the FCOA except when the Gland 2 image threshold is 2. To be exact, the PSNR value of the ISBO is 0.8635 higher than that of the FCOA, with an average increase ratio of 4.96%. The highest increase occurs when the threshold of Brn2 image is 2, with an increase of 4.1066, and an increase ratio of 23.57%.

### Comparison and Analysis of Plant Image Segmentation Experiments

**Figure 3:**
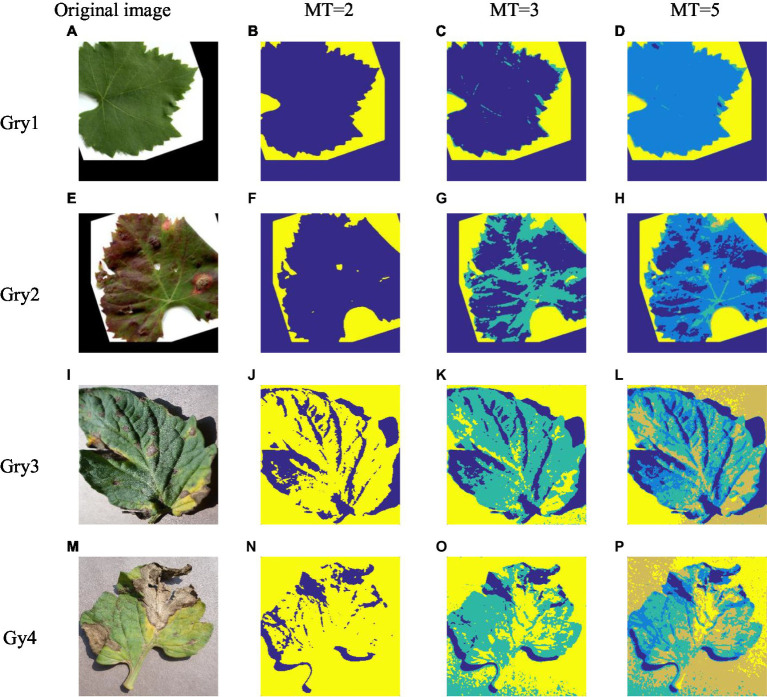
The plant image segmentation result based on ISBO.

In order to verify the superiority of ISBO and fully reflect its practical application value, we apply this algorithm to the plant image segmentation. In our study, four plant images in the Kaggle plant image dataset^3^ are selected for experiment, and the parameter setting is consistent with that discussed in “Parameter Setting and Discussion” section. Figures 3B–D, F–H, J–L and N–P show the image segmentation results of the four plant images (Figures 3A,E,I,M) by ISBO with different threshold numbers. The corresponding Table 8 lists the experimental result data.

**Table 8:** Experimental data of the plant image segmentation.

Image	MT	Thresholds					PSNR	FSIM
Gry1	2	105	187				16.2897	0.7236
	3	82.5	123.5	183.5			16.6751	0.7703
	5	27.5	76	144.5	178.5	226.5	26.1164	0.8181
Gry2	2	112	197				16.5825	0.6447
	3	51	101	175			19.0693	0.6779
	5	36	83.5	125	182	225.5	22.1451	0.7327
Gry3	2	43.5	135.5				16.4277	0.5099
	3	37	117	194.5			19.2348	0.5989
	5	37	92.5	125	165	232.5	20.7436	0.7151
Gry4	2	66.5	180.5				20.1022	0.5137
	3	59	138.5	185.5			22.0812	0.6039
	5	23.5	106.5	150	171	200.5	23.5093	0.7078

Figure 3 shows that ISBO achieves more refined plant image segmentation, and effectively realizes the segmentation of lesion area. Like medical image analysis, it cannot effectively make the comparative analysis with other similar algorithms in a visual way. Therefore, Table 8 lists the threshold distribution and PSNR and FSIM quantitative data corresponding to the plant image segmentation. Table 8 shows that with the increase in the number of thresholds, the threshold vectors are more evenly distributed; the values of PSNR and FSIM are higher, thus proving that the proposed algorithm in our work can effectively realize the segmentation of plant disease images.

To more intuitively evaluate the advantages and disadvantages of ISBO in plant image segmentation, this section also compares ISBO with SBO, FGWO and FCOA. Table 9 lists the experimental result data of the four algorithms. It shows that the PSNR value of ISBO is superior to that of SBO in all cases. Specifically, compared with SBO, the PSNR value of the ISBO increases by 0.6719 on average, and the average increase percentage is 3.49%. And the maximum increase reaches 3.5591 and the minimum increase reaches 0.0172. By percentage, the maximum increase reaches 18.5%, the minimum 0.09%. Compared with the FGWO, the ISBO is superior under other conditions except when the threshold of Gry3 is 5. Specifically, the PSNR value of the ISBO is 1.4126 higher than that of the FGWO, and the average increase percentage is 7.67%. In the case of the Gry3 image when the exceptional threshold 5 is set, the PSNR value of the ISBO is only 0.0607 lower than that of the FGWO. Finally, compared with the FCOA, the ISBO still obtains higher PSNR value in all cases. Similarly, compared with the FCOA, the PSNR value of the ISBO increases by 1.5146 on average, with an average increase percentage of 8.23%, of which the highest and lowest increase percentages are 51.3% and 0.05%, respectively.

**Table 9:** Comparison of experimental data of the plant images.

Image	MT	PSNR			
		ISBO	SBO	FGWO	FCOA
Gry1	2	16.2897	16.2725	16.2854	16.2804
	3	16.6751	16.3979	16.5253	16.5139
	5	26.1164	26.0164	16.8329	16.6779
Gry2	2	16.5825	16.5326	16.5798	16.5732
	3	19.0693	16.9781	17.4758	17.1470
	5	22.1451	18.5860	18.7410	18.6002
Gry3	2	16.4277	16.3589	16.4068	16.0568
	3	19.2348	18.9205	18.7574	17.9570
	5	20.7436	20.5342	20.8043	20.6044
Gry4	2	20.1022	19.8106	20.0157	19.9443
	3	22.0812	21.4377	21.9520	21.8896
	5	23.5093	23.0684	23.1221	22.5566

## Conclusion

In order to fully verify the value of the intelligent optimization algorithm based on specific objective function in medical and plant image segmentation applications, this paper introduces the ISBO into the multi-level thresholding of medical and plant images. The algorithm takes fuzzy Kapur as the objective function and optimizes a set of thresholds by improving the SBO algorithm to complete the initial image segmentation. On this basis, the median aggregation method is introduced to avoid the problem of over-segmentation or segmentation of outliers. In the process of improving the SBO algorithm, chaos initialization and Cauchy mutation strategy are also introduced to improve the convergence speed of the algorithm and reduce the risk of falling into local optimization. To prove the superiority of ISBO, this paper compares ISBO with SBO, FGWO and FCOA using medical and plant images. Through the comparison of visual and quantitative data, it can be observed that ISBO is more effective in the segmentation of medical and plant images.

## Data Availability Statement

The original contributions presented in the study are included in the article/supplementary material, further inquiries can be directed to the corresponding author.

## Author Contributions

All authors listed have made a substantial, direct, and intellectual contribution to the work and approved it for publication.

## Funding

This paper is supported by the National Youth Natural Science Foundation of China (61802208), the National Natural Science Foundation of China (61572261), the Natural Science Foundation of Anhui (1908085MF207, KJ2020A1215, KJ2021A1251, and KJ2021A1253), the Excellent Youth Talent Support Foundation of Anhui (gxyqZD2019097 and gxyqZD2021142), the Postdoctoral Foundation of Jiangsu (2018K009B), and the Foundation of Fuyang Normal University (TDJC2021008).

## Conflict of Interest

The authors declare that the research was conducted in the absence of any commercial or financial relationships that could be construed as a potential conflict of interest.

## Publisher’s Note

All claims expressed in this article are solely those of the authors and do not necessarily represent those of their affiliated organizations, or those of the publisher, the editors and the reviewers. Any product that may be evaluated in this article, or claim that may be made by its manufacturer, is not guaranteed or endorsed by the publisher.

## Footnotes

^1^ https://brainweb.bic.mni.mcgill.ca/brainweb/

^2^ https://warwick.ac.uk/fac/cross_fac/tia

^3^ https://www.kaggle.com/datasets/asheniranga/leaf-disease-dataset-combination
